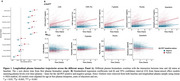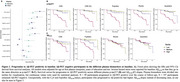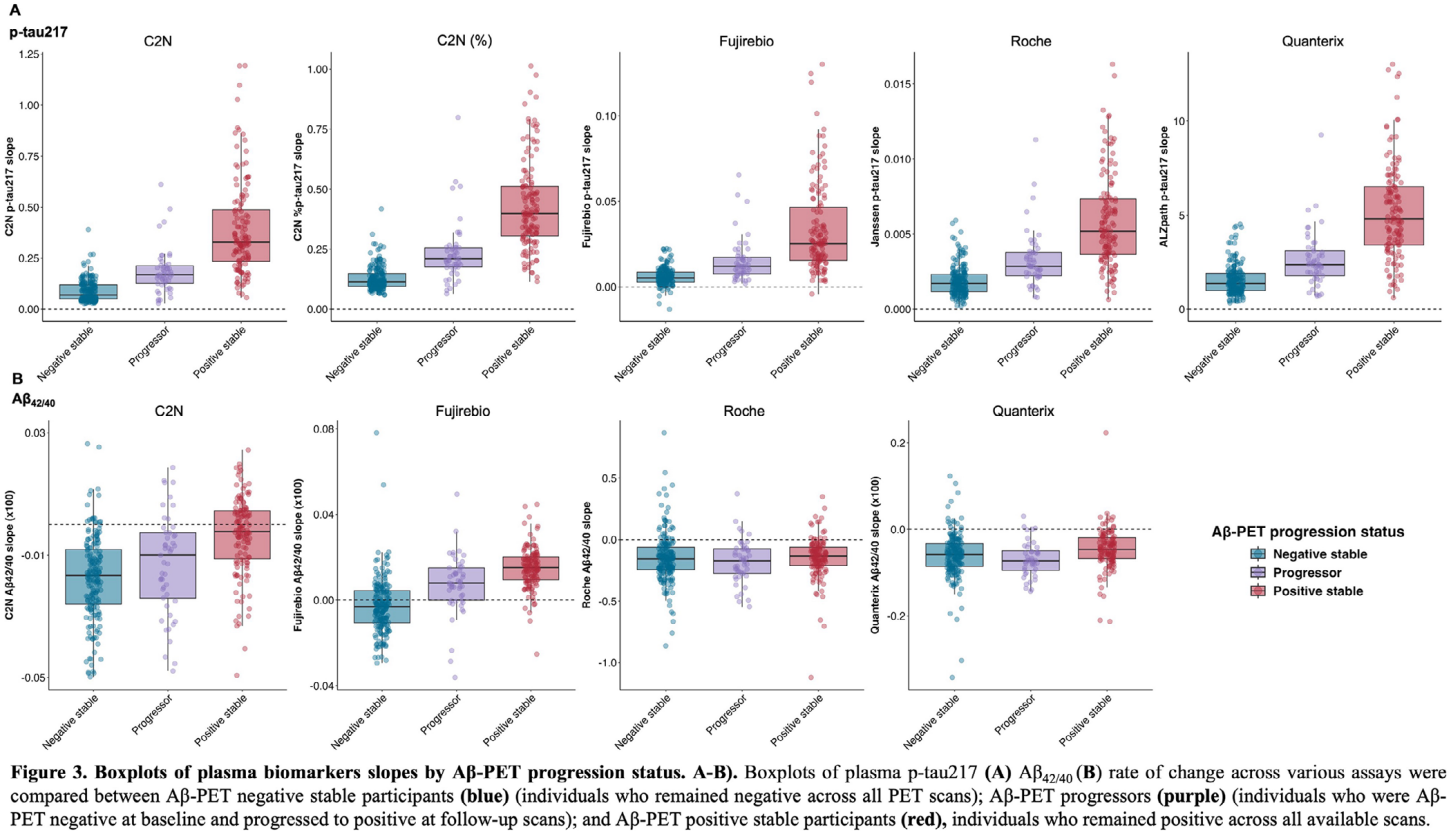# Comparison of Longitudinal Plasma Assays in Relation to Longitudinal Amyloid Pathology in Alzheimer's Disease

**DOI:** 10.1002/alz70856_105944

**Published:** 2026-01-07

**Authors:** Yara Yakoub, Ting Qiu, Sylvia Villeneuve, Alexa Pichet Binette, Alzheimer's Disease Neuroimaging Initiative (ADNI)

**Affiliations:** ^1^ Integrated Program in Neurosciences, McGill University, Montréal, QC, Canada; ^2^ Douglas Mental Health University Institute, Centre for Studies on the Prevention of Alzheimer's Disease (StoP‐AD), Montréal, QC, Canada; ^3^ Department of Psychiatry, McGill University, Montréal, QC, Canada; ^4^ Department of Physiology and Pharmacology, Université de Montréal, Montréal, QC, Canada; ^5^ Clinical Memory Research Unit, Department of Clinical Sciences Malmö, Faculty of Medicine, Lund University, Lund, Sweden; ^6^ Centre de Recherche de l’Institut Universitaire de Gériatrie de Montréal, Montréal, QC, Canada

## Abstract

**Background:**

Blood‐based biomarkers of AD, especially *p*‐tau217, show high concordance with Aβ‐PET load and status. While many studies relied on cross‐sectional data, assessing dynamic changes in these assays is important for future clinical use and trial outcomes. We evaluated the longitudinal trajectories of multiple plasma biomarkers and their associations with subsequent changes in Aβ‐PET status.

**Method:**

We used data from the ADNI FNIH consortium consisting of 386 participants (72.7 ± 7.1 years old, 52.3% cognitively unimpaired, 49.7% women) with on average three plasma samples and three Aβ‐PET scans collected over 11 years, in which multiple assays were tested (here focussing on 5 *p*‐tau217 and 4 Aβ_42/40_ assays). We first assessed changes over time for each assay in Aβ‐PET positive (*n* = 140, 36.3%) and negative participants. We then focused on progression from Aβ‐negativity to Aβ‐positivity over follow‐up, using cox proportional‐hazard models to evaluate if baseline levels and longitudinal (rate of change) of plasma biomarkers were related to subsequent Aβ‐positivity (total of 49 progressors).

**Result:**

Across both Aβ‐negative and positive groups, all *p*‐tau217 assays, but not the Aβ_42/40_ assays, showed increase over time (Figure 1A‐B). In the Aβ‐negative subgroup, higher plasma *p*‐tau217 rate of change was associated with increased risk of progression to Aβ‐PET positivity (Figure 2A‐B), with highest hazard rations (HR) seen with the C_2_N assays (HR_p‐tau217_ = 2.77 and HR_p‐tau217 %_ = 2.50). Hazard ratios were higher with *p*‐tau217 rates of change compared to baseline values (Figure 2A). With the Aβ_42/40_ assays, higher Aβ_42/40_ pathology at baseline was associated with progression to Aβ‐PET positivity (HR from 1.44 ‐1.65), but no associations or unexpected associations were seen with the rate of change (Figure 2A). Individual slopes between the different groups (Aβ‐negative, progressors to Aβ‐positive and Aβ‐positive) for all assays are displayed in Figure 3 to aid in assay comparisons.

**Conclusion:**

All plasma *p*‐tau217 assays levels changed over time, which was not the case for the Aβ_42/40_ assays. Among Aβ‐negative individuals, while baseline plasma *p*‐tau217 and Aβ were associated with progression to Aβ‐positivity over 10 years, across all assays, the rate of change in *p*‐tau217 was the measure most strongly predictive of future Aβ‐PET positivity.